# Cigarette smoking as a risk factor for ischaemic stroke in young South Asian male migrants to Qatar: The BRAINS study

**DOI:** 10.5339/qmj.2023.23

**Published:** 2023-12-12

**Authors:** Fahmi Yousef Khan, Gie Ken-Dror, Paul Ly, Hassan Al Hail, Dirk Deleu, Musab Ali, Hassan Al Hussein, Hassan Osman Abuzaid, Khalid Sharif, Pankaj Sharma

**Affiliations:** ^1^Hamad Medical Corporation, Doha, Qatar.; ^2^Institute of Cardiovascular Research, Royal Holloway University of London (ICR2UL), United Kingdom Email: pankaj.sharma@rhul.ac.uk https://orcid.org/0000-0003-3641-7441; ^3^Department of Neurology, Neuroscience Institute, Hamad Medical Corporation, Doha, Qatar.; ^4^Imperial College Healthcare NHS Trust, London UK.

**Keywords:** Ischaemic stroke, cigarette smoking, South Asian, migrants

## Abstract

Background: The incidence of stroke in the Middle East is high, given its relatively young population. Smoking is a well-recognized risk factor for ischaemic stroke, and its high regional prevalence may partly account for this increased stroke risk. This research aims to determine whether young male South Asian migrants in Qatar were adversely affected by stroke depending on their smoking status.

Methods: Data from the ongoing international prospective BRAINS study was analysed. Male South Asian migrants to Qatar with a history of ischaemic stroke were recruited. Multivariate regression analysis was used to estimate the effects of comorbidities, such as BMI, hypertension, diabetes, hypercholesterolemia, alcohol consumption, and ischemic heart disease, on the association of age of stroke onset and smoking status.

Results: We identified 778 (mean age 49.5±10.2) migrant male workers of South Asian descent with ischaemic stroke in Qatar, of which 41.3% of the sample were current smokers. Compared to non-smokers, current smokers suffered a stroke 2.03 years earlier (95%CI: 0.60–3.46, *P*=0.005). Multivariate regression analysis demonstrated that only current smoking status was associated with an earlier age of stroke onset (β=2.03, SE=0.74, *P*=0.006).

Conclusion: Smoking is associated with at least a two-year earlier onset of ischaemic stroke in male South Asian migrants to the Middle East. Our study has important implications for the public health management of migrants in host countries.

## Introduction

Qatar hosts over 2.71 million residents, with only ~13% being Qatari citizens.^1,2^ About 27.6% of the population are females, while 72.4% are males.^2^ Most migrants are attracted by the oil and gas industry leading to rapid urbanization.^3^ Correspondingly, low-skilled working migrants^3^, mainly of South Asian or North African descent, form a large part of migrant employees.^4^ Previous studies have suggested that South Asian migrants show an increased risk of developing vascular diseases compared to native residents of the host country^5^, which may be attributed to exposure to local changes in lifestyle, five or a genetic predisposition.^6,7^ A cross-sectional study of Indian subcontinental migrant workers in Qatar showed that they suffer from high rates of diabetes, hypertension, and cardiopulmonary diseases.^4^

Stroke is a significant cause of mortality in Qatar.^8^ In an observational study in Qatar, smoking was one of the main risk factors found in young adults admitted with ischaemic stroke,^9^. However, the migrant population has not been well studied. Hence, we sought to determine whether smoking hurt stroke onset in South Asian males who migrated to Qatar in this study.

## Methods

The Biorepository of DNA in Stroke (BRAINS) is an ongoing international prospective study for stroke patients recruited from the UK, India, Qatar, and Sri Lanka. The detailed protocol of BRAINS^10^ and its enrolment in Qatar has previously been published.^11^ Briefly, the study recruits stroke patients mainly of South Asian ancestry. Only subjects with magnetic resonance imaging (MRI) / computerized tomography (CT)) confirmed stroke are recruited. Extensive demographic and clinical details are documented, including the history of smoking, alcohol use, diabetes, hypertension, body mass index (BMI), glucose level, and hypercholesterolemia measurements. Ethnic and religious data, as well as the country of origin (Indians, Bangladeshis, Nepalese, Pakistanis, Filipinos, Sri Lankans) based on grandparent origin, are reported. For this work, South Asian males who migrated to Qatar and suffered an ischaemic stroke were studied. Most migrants to Qatar are males, so our analysis focused on this gender. There were too few South Asian females with stroke to provide meaningful results.

A trained clinical nurse self-reported the smoking status on a detailed questionnaire. Patients were categorized into non-smokers or current smokers. Non-smokers were defined as those who had never smoked any tobacco product. Current smokers were defined as those who smoked any tobacco product during recruitment. Ex-smokers were not included in the study.

### Statistical Analysis

Descriptive statistics were summarized using mean with standard deviation (SD) for continuous variables and proportion for categorical variables. The chi-square or Fisher’s exact test was used for single factor analysis of categorical variables. Independent t-test or Mann-Whitney U test was used for single factor analysis of continuous variables. A one-way analysis of covariance (ANCOVA) was conducted to compare the differences in age of stroke onset among smoking status controlling for traditional risk factors (BMI, hypertension, diabetes, hypercholesterolemia, ischemic heart disease (IHD), and alcohol consumption). Normality checks and Levene’s test were carried out. To evaluate the association of smoking status with the age of stroke onset in South Asian migrants, a linear regression model was performed, adjusted for the following variables: BMI, hypertension, diabetes, hypercholesterolemia, IHD, and alcohol consumption. Regression coefficients (Beta) were calculated for independent risk factors. Stepwise regression analysis with Akaike’s information criterion (AIC),^12,13^ including potential confounders, was performed to identify the independent determinants of the age of stroke onset and those with the most substantial contribution to overall variance. Analyses were performed and produced using R *v*4.0.2, with *P*<0.05 taken to be statistically significant throughout.

## Results

The study population was 778 migrant male workers of South Asian descent (Indians, Bangladeshis, Nepalese, Pakistanis, Filipinos, Sri Lankans) with ischaemic stroke. The prevalence of hypertension was 75.1% (95%CI: 71.9–78.1), diabetes 46.0% (95%CI: 42.5–49.6), hyper­cholesterolemia 40.0% (95%CI: 36.5–43.5), and alcohol 31.7% (95%CI: 28.5–35.4), was calculated. The mean age was 49.5±10.2 years, and 41.3% (95%CI: 37.8–44.8) of the sample were current smokers.

Demographic and clinical characteristics of smoking status are presented in Table 1. Current smokers had a lower average BMI value than non-smokers by 0.92 kg/m^2^ (95%CI: 0.37–1.47, *P*=0.001). No significant differences were observed in nationality groups (*P*=0.52), hypertension (*P*=0.11), diabetes (*P*=0.58), hypercholesterolemia (*P*=0.75), and IHD (*P*=0.79) levels between current smokers and non-smokers. However, current smokers had a higher prevalence of alcohol consumption by 20.0% (95%CI: 13.2–26.8, *P*<0.001) compared with non-smokers. Moreover, it was observed that current smokers suffered stroke 2.03 years earlier (95%CI: 0.60–3.46, *P*=0.005), compared to non-smokers.

A one-way ANCOVA was conducted to compare the differences in age of stroke onset among smoking status controlling for traditional risk factors (BMI, hypertension, diabetes, hypercholesterolemia, IHD, and alcohol consumption). Normality checks and Levene’s test were carried out, and the assumptions were met. There was a significant difference in mean age (*P*=0.006) between the smoking status. There was a significant difference between current and non-smokers (*P*=0.006), with current smokers 2.03 years (95%CI: 0.57–3.48) younger than non-smokers. Comparing the estimated adjusted means showed that current smokers were the youngest group (48.1 years, 95%CI: 46.2-49.9) compared to non-smokers.

To evaluate the association of smoking status with the age of stroke onset in South Asian migrants, a linear regression model was performed, adjusted for the following variables: BMI, hypertension, diabetes, hypercholesterolemia, IHD, and alcohol consumption (Table 2). In multiple linear regression, current smokers compared to non-smokers were associated with an earlier age of onset (β=-1.98, SE=0.77, *P*=0.01). Following a stepwise linear regression model selection by AIC that was performed adjusting for traditional stroke risk factors, current smokers were the only independent predictor showing a negative association with age of onset of stroke (β=2.03, SE=0.74, *P*=0.006).

## Discussions

Using an ongoing large international stroke study, we show that ischaemic stroke occurs two years earlier in young South Asian male migrant smokers compared to non-smokers. This result was independent of other traditional risk factors (BMI, hypertension, diabetes, hypercholesterolemia, IHD, and alcohol consumption).

According to a meta-analysis of 14 studies and 303,134 subjects, current smokers had an overall increased risk of stroke compared to non-smokers, with a pooled OR of 1.92 (95%CI: 1.49–2.48).^14^ Smoking increases the development of atherosclerosis and the concentration of low density,^15^ leading to hypercholesterolemia.^16^ In addition, smokers have a higher risk of atrial fibrillation.^17^ Further, hypertensive smokers are at higher risk for ischaemic stroke since their arteries narrow quicker.^18^

Significant clinical characteristic differences (BMI, alcohol consumption, hypertension, high cholesterol, and diabetes) among South Asian stroke migrants separated by smoking status. Current smokers had ~1 kg/m^2^ lower BMI values, which is unsurprising as smoking is known to be associated with weight loss, likely due to increased energy expenditure and reduced appetite.^19^ Conversely, smoking cessation can lead to an increased risk of diabetes.^20^ Further, cigarette smoking can stimulate the sympathetic nervous system, which may produce a hypertensive effect.^21^ Moreover, 20% of smokers more frequently described themselves as regular drinkers. Alcohol drinkers have an elevated risk of ischaemic stroke for ~2 hours following consumption of a single serving.^22^ Moreover, alcohol is capable of causing atrial fibrillation, which can cause blood clots that lead to ischaemic stroke.^17,18,23^ South Asian populations tend to have strokes earlier than the Caucasian population, with higher levels of hypertension, high cholesterol, and diabetes.^24−27^ Our study supports these results among South Asian smoking migrant male workers in Qatar.

It has been widely established that smoking or using tobacco products increases the risk of ischemic stroke.^14,28,29^ Smoking is not widely popular among South Asian youths and females, but it is accepted at social events and is becoming increasingly popular.^30^ In addition, certain populations are unaware of the negative health effects of smoking.^31^ Bangladeshi men had the highest percentage of current smokers (40%), followed by Pakistani (29%) and Indians (20%) when specific ethnic groupings were broken down by gender.^30^ There are several different tobacco-based products that South Asians utilize, including chewing tobacco, pipe tobacco, chukka, chutta, chillum, and bidis.^32^ These tobacco products typically include large amounts of tar and nicotine.^32^ Among the Bangladeshi population, chewing tobacco is the most frequent form of use, with 9% of males and 16% of females using this practice.^30^ Under-reporting of tobacco use has been a significant issue for both types of tobacco use, making its impact difficult to measure. Despite the decreased prevalence reported among South Asians, it should be noted that most studies rely on self-reported use of tobacco.^30,33,34^

### Limitations

Several limitations need to be considered when assessing this work. The majority of a subgroup of South Asians were of Indian origin rather than of Pakistani, Sri Lankan, or Bangladeshi origin. A subgroup analysis by ethnic groups did not affect the results, but some sample sizes were too small for analysis. More significant numbers in these populations may have demonstrated differences in subgroups. All our subjects were male. There are few South Asian female migrants in Qatar and even fewer female smokers. Thus, our results cannot be extended to females. The categorization of smoking status was based on self-reported information, and ex-smokers were not included in our study. While possible that self-reporting can be open to error, most patients were keen to understand the cause of their stroke, mainly as they were young and in a foreign land. Passive smoking^35^ was not accounted for and could be a significant confounding factor among non-smokers. Our results cannot be extrapolated to other migrant workers as the environment to which such workers are exposed and the healthcare facilities and accessibility to such care will differ in each location.

## Conclusions

In Qatar, young South Asian male migrants who smoke are likely to suffer an ischaemic stroke at least two years earlier than non-smokers. In addition, there were significant clinical characteristic differences (BMI, alcohol consumption, hypertension, high cholesterol, and diabetes) among South Asian stroke migrants separated by smoking status. Our study has implications for public health management in host countries of migrant workers. Such countries should target their anti-smoking campaigns to migrant workers in their languages to have maximum effect.

## Clinical Trial Registration

URL: http://www.clinicaltrials.gov.

Unique identifier: NCT05491824.

## Funding

The global BRAINS study has received funding from the Henry Smith charity and is supported by the UK Clinical Research Network. PS was supported by a personal Clinical Fellowship from the Department of Health during part of this study. The Qatar arm of BRAINS was funded by grants from the Qatari National Research Fund (NPRP 6-068-3-015).

## Declaration of Interest Statement

The authors declare that no conflict of interest could be perceived as prejudicing the impartiality of the research reported.

## Data Availability Statement

No data are available.

## Ethical Approval

Ethical approval for this study was obtained from Riverside Research Ethics Committee (04/Q0401/40). Research governance is through the standard Royal Holloway University of London (RHUL) procedures and protocols. Ethical approval was also received from Hamad Medical Corporation (RC/66412/2012 and JIRB 14-00064).

Clinical Trial Registration: URL: http://www.clinicaltrials.gov. Unique identifier: NCT05491824.

## Abbreviations

BRAINS: Biorepository of DNA in Stroke; SD: standard deviation; GLM: General linear model; AIC: Akaike’s information criterion; IHD: Ischemic Heart Disease; CI: Confidence Intervals; BMI: Body Mass Index; ANCOVA: Analysis of covariance; SE, standard error.

## Figures and Tables

**Table 1.** Demographic and clinical characteristics for all South Asian stroke male migrants by smoking status in Qatar.

	Current Smoker (n=321)	Non-Smoker (n=457)	*P*-value
Nationality, n (%)			
Indians	111 (37.5)	185 (62.5)	0.52
Bangladeshis	74 (47.7)	81 (52.3)	
Nepalese	38 (41.3)	54 (58.7)	
Pakistanis	33 (38.4)	53 (61.6)	
Filipinos	29 (44.6)	36 (55.4)	
Sri-Lankans	23 (42.6)	31 (57.4)	
Other	13 (44.8)	16 (55.2)	
**BMI (kg/m^2^)**	26.1 ± 3.8	27.0 ± 4.0	0.001
**Alcohol consumption, n (%)**	129 (40.2%)	92 (20.2%)	<0.001
**Comorbidities, *n* (%)**			
Hypertension, n (%)	231 (72.0%)	353 (77.2%)	0.11
Diabetes, n (%)	152 (47.4%)	206 (45.1%)	0.58
Hypercholesterolemia, n (%)	108 (33.6%)	160 (35.0%)	0.75
Ischemic Heart Disease, n (%)	18 (5.6%)	29 (6.3%)	0.79
Atrial Fibrillation, n (%)	2 (0.6%)	2 (0.4%)	0.99
**Age of stroke onset (years, SD)**	48.3 ± 9.6	50.4 ± 10.5	0.005

n, sample size; BMI, body mass index; SD, standard deviation.

**Table 2.** Multivariate linear regression analysis of South Asian stroke male migrants age (years) of ischemic stroke onset with traditional risk factors.

Predictor	Effect size (*β*), years	SE	*t*-value	*P*-value
**Smoking status (Non-Smoker reference group)**				
**Current Smoker**	**-1.98**	**0.77**	**-2.58**	**0.01**
BMI (kg/m^2^)	-0.01	0.09	-0.04	0.97
Alcohol consumption (Yes)	-0.23	0.84	-0.27	0.78
Hypertension (Yes)	0.59	0.87	0.68	0.50
Diabetes (Yes)	0.97	0.75	1.29	0.20
Hypercholesterolemia (Yes)	-0.87	0.79	-1.11	0.27
IHD, (Yes)	-0.06	1.56	-0.04	0.97

* Effect size (β-coefficients), degree of change in the age of onset of Ischemic stroke (dependent variable) for every 1 unit of change in the predictor variable. SE, standard error; BMI, body mass index; IHD, Ischemic Heart Disease.
